# Increased proliferation and altered cell cycle regulation in pancreatic stem cells derived from patients with congenital hyperinsulinism

**DOI:** 10.1371/journal.pone.0222350

**Published:** 2019-09-16

**Authors:** Sophie G. Kellaway, Karolina Mosinska, Zainaba Mohamed, Alexander Ryan, Stephen Richardson, Melanie Newbould, Indraneel Banerjee, Mark J. Dunne, Karen E. Cosgrove

**Affiliations:** 1 Faculty of Biology Medicine and Health, School of Medicine, Division of Diabetes Endocrinology and Gastroenterology, University of Manchester, Manchester, United Kingdom; 2 Department of Paediatric Endocrinology, Royal Manchester Children’s Hospital, Manchester, United Kingdom; 3 Department of Paediatric Histopathology, Royal Manchester Children’s Hospital, Manchester, United Kingdom; Odense University Hospital, DENMARK

## Abstract

Congenital hyperinsulinism (CHI) is characterised by inappropriate insulin secretion causing profound hypoglycaemia and brain damage if inadequately controlled. Pancreatic tissue isolated from patients with diffuse CHI shows abnormal proliferation rates, the mechanisms of which are not fully resolved. Understanding cell proliferation in CHI may lead to new therapeutic options, alongside opportunities to manipulate β-cell mass in patients with diabetes. We aimed to generate cell-lines from CHI pancreatic tissue to provide *in vitro* model systems for research. Three pancreatic mesenchymal stem cell-lines (CHIpMSC1-3) were derived from patients with CHI disease variants: focal, atypical and diffuse. All CHIpMSC lines demonstrated increased proliferation compared with control adult-derived pMSCs. Cell cycle alterations including increased CDK1 levels and decreased p27^Kip1^ nuclear localisation were observed in CHIpMSCs when compared to control pMSCs. In conclusion, CHIpMSCs are a useful *in vitro* model to further understand the cell cycle alterations leading to increased islet cell proliferation in CHI.

## Introduction

Congenital hyperinsulinism (CHI) presents in the neonatal period or early infancy and is associated with profound hypoglycaemia due to high levels of unregulated insulin secretion [1]. There are three histological forms of CHI—focal, diffuse and atypical. Focal CHI is most commonly due to a recessive mutation in the *ABCC8* gene, where loss of heterozygosity leads to no functional allele and non-functional K_ATP_ channels [2–4]. This form is named for the fact it only affects a focal lesion within the pancreas which is almost exclusively enriched by β-cells. The loss of heterozygosity also affects the cyclin-dependent kinase inhibitor (CKI) p57^Kip2^, a likely contributor to β-cell hyperplasia seen in focal CHI [4, 5]. Diffuse CHI is usually due to a homozygous recessive mutation in one of a number of different genes, including *ABCC8* and every β-cell within the pancreas is affected [6, 7]. Atypical CHI usually has a later onset than focal or diffuse CHI, is not caused by any known germline mutation (screening of the genes associated with focal and diffuse CHI excludes these) and leads to mosaicism of affected islet cells [8]. It has also been shown that atypical CHI is associated with altered expression of hexokinase and NKX2.2 in some individuals [9, 10].

We recently described abnormal proliferation of a range of different pancreatic cell types in diffuse CHI patients compared to age-matched controls, as documented by the number of Ki67 positive cells, which may be a factor in the disease pathology [11, 12]. This was found to be associated with a high number of islet-cell nuclei containing CDK6 and p27^Kip1^ [12]. CDK6 and p27^Kip1^ are cell cycle regulators involved in the G_1_/S transition. The progression through the G_1_/S checkpoint commits a cell to division and alterations to cell cycle regulators can therefore affect the proliferation rates of cells [13]. The cell cycle is controlled by a multitude of both positive and negative regulators including cyclins, cyclin dependent kinases (CDKs) and CKIs, with many proteins showing sequence similarities, multiple roles, and functional redundancy [14, 15].

Understanding the factors underpinning islet cell proliferation in CHI may ultimately be of use for islet regeneration and stem cell therapies for diabetes, but opportunities to study CHI tissue are limited due to CHI being a rare human disease with few opportunities to access surgery derived pancreatic tissue. Studies on fixed post-operative CHI tissue provide useful but static information without scope to manipulate *in vivo* experimental conditions to generate dynamic data. Whilst rodent models of CHI have been generated, β-cell duplication occurs in the rodent pancreas specifically [16], so does not accurately represent events occurring in human pancreas, meaning that this proliferative element of CHI cannot be optimally replicated [17, 18]. There are also differences in the cell cycle regulators used by rodents and humans, the cell cycle is well studied in rodents but there are several elements such as the usage of CDK4/6 and cyclinD2 which are not applicable or unclear in humans [18, 19].

The aim of the present study was to generate cell lines from CHI pancreatic tissue which could be used as *in vitro* cell models to study proliferation and cell cycle regulation. Here we describe the generation of a panel of three pancreatic mesenchymal cell lines from pancreatic CHI tissue, designated CHIpMSC1-3, and their subsequent use to explore regulation of p27^Kip1^ at varying glucose and insulin concentrations.

## Materials and methods

### Derivation of CHI pancreatic mesenchymal stem cell (CHIpMSC) lines

Pancreatic tissue from three patients undergoing pancreatectomy for intractable hypoglycaemia was collected with ethical permission and informed consent and used to generate cell lines (Table 1). Tissue was rinsed in Krebs-Ringer-HEPES buffer containing 0.1% BSA and 5.6 mM glucose (KRH buffer) to remove excess blood. The tissue was digested with 0.75 mg/ml Liberase (Roche, Mannheim, Germany) in KRH buffer with 1.5 mg/ml egg white trypsin inhibitor (Sigma-Aldrich, Irvine, UK) and 1.5 mg/ml soybean trypsin inhibitor (Gibco, Life Technologies, Paisley, UK), for 3 minutes at 37°C with agitation, followed by 1 minute of vigorous shaking, and this was done three times. Ice cold KRH buffer was added and the digestion mixture centrifuged for 1 minute at 150 x g. The secondary digestion was with 0.5 mg/ml Liberase in KRH buffer with trypsin inhibitors, for 3 minutes at 37°C with agitation, followed by 1 minute of vigorous shaking, and this was done twice. Ice cold KRH buffer was added and the digestion mixture centrifuged for 1 minute at 150 x g, this was done 4 times to ensure the preparation was as clean as possible. The resulting cells and tissue fragments comprising mixed islet cells and exocrine tissue were transferred to a 6-well tissue culture treated plate in RPMI-1640 5.5 mM glucose (Sigma-Aldrich/Gibco) with 10% foetal bovine serum (FBS, Gibco), roughly 1 well was used per 20 mg wet weight tissue received. After 48 hours, cells and tissue which had not adhered were removed and cells were cultured as described in Section 2.3. Authenticity and stability of cell lines was confirmed by short tandem repeat profiling, genotyping for the disease-causing mutation and karyotyping.

**Table 1:** Details of CHI pancreatic tissue used to generate cell lines.

Patient number	Diagnosis	Gene Mutation	Age at surgery	Cell line
#1	Focal CHI	Heterozygous *ABCC8* exon 6 c.1016G>A; p.Gly316Arg	4 months	CHIpMSC1
#2	Atypical CHI	Not known	17 months	CHIpMSC2
#3	Diffuse CHI	Homozygous *ABCC8* exon 37 c.4481G>A; p.Arg1494Gln	11 weeks	CHIpMSC3

Tissue from Patient #1 pancreatic tissue was taken adjacent to the focal lesion therefore the expected genotype was heterozygous. Patient #2 was screened for mutations in *ABCC8* and *KCNJ11*. Additional genetic analysis was performed by testing DNA samples extracted from pancreatic tissue following surgery by examining the coding regions and exon/intron boundaries of the *ABCC8*, *KCNJ11*, *HNF4A*, *HADH*, *GCK*, *GLUD1*, *INSR*, *SLC16A1*, *TRMT10A* and *HNF1A* genes by targeted next-generation sequencing to high depth (mean coverage across genes: 613x).

### Derivation of adult pancreatic mesenchymal stem cell (pMSC) lines

Purified islets from adult organ donors were received from the Diabetes Research Wellness Foundation Human Islet Isolation Facility, University of Oxford, when preparations were of insufficient purity or viability for transplantation and donors had given consent for the tissue to be used for research. The islets were directly placed into 6-well tissue culture-treated plates at a density of approximately 100 islets per well, and then treated as per the CHIpMSC lines.

### Derivation of bone marrow mesenchymal stem cell (bmMSC) lines and RNA extraction

Bone marrow was obtained from the proximal femur of three donors (ages 48–75 years; mean age 59 years; all male) undergoing hip replacement surgery after approval from the National Research Ethics Service and fully informed written consent of patients. MSCs were isolated using density gradient centrifugation with RosetteSep (Stem Cell Technologies Inc) blood cell removal and plastic adherence, and total RNA was extracted from P3-4 cells using TRIzol reagent, all as previously described [20].

### Cell culture

Cells were routinely cultured on tissue culture plastic (plates or vented cap flasks, Corning, Schipol-Rijk, Netherlands) coated with human fibronectin (Chemicon, Merck Millipore, Nottingham, UK) to 0.5 μg/cm^2^ and vitronectin (Life Technologies) to 0.1 μg/cm^2^, in RPMI-1640 5.5 mM glucose with 10% FBS. Cultured cells were kept in a humidified incubator at 37°C, 5% CO_2_. Passage was performed when cells reached approximately 70% confluency and were seeded at a cell line and condition specific density, between 1:2 and 1:5. Culture medium was changed every 48 hours. For growth curves, 2 x 10^2^ cells were seeded per well, in uncoated 24-well plates and counted using trypan blue exclusion on a haemocytometer. For cumulative cell population data, uncoated plastic was also used. Low glucose culture was in 2 mM glucose RPMI-1640 with 10% FBS; high insulin culture was in RPMI-1640 with 10% FBS (38 nM insulin determined by ELISA) supplemented with 200 nM insulin (Life Technologies). All experiments were performed on CHIpMSCs between passages 5 and 14, and on adult pMSCs between 4 and 9 and at least one passage prior to growth arrest.

### Gene expression analysis

Total RNA was extracted from CHIpMSCs and bone marrow stem cells using an RNeasy Mini Kit (Qiagen) with on column DNase 1 (Qiagen) genomic DNA digestion according to the manufacturer’s instructions and quantified using a NanoDrop 1000 spectrophotometer. cDNA was synthesised from 500 ng RNA using a nanoScript 2 reverse transcription kit (Primer Design) with oligo-dT primers and random nonamer primers. qRT-PCR was carried out using power SYBR green PCR master mix (Life Technologies) and 2.5 pmol of each primer and run on a StepOnePlus Real-Time PCR System (Life Technologies). The CT values of the gene of interest were normalised to the geomean of 2 reference genes (ΔCT) and anti-logged (2^-^ΔCT). Where samples were “undetermined” they were considered as 50, which was the maximum number of cycles run. The TaqMan gene expression assays used were as follows, all FAM-MGB: ISL1 Hs00158126_m1, B2M Hs99999907_m1 and PPIA Hs99999904_m1.

### Adipogenesis, osteogenesis and chondrogenesis

Cells were cultured in StemPro Adipogenesis, Osteogenesis or Chondrogenesis media (Gibco) as per the manufacturer’s instructions, with chondrogenesis taking place as a micromass culture. After 14 days for adipogenesis and chondrogenesis or 21 days for osteogenesis in these conditions, cells were fixed in 4% paraformaldehyde. Lipid droplets were stained with oil red O (0.3% w/v; Sigma-Aldrich) following incubation with 60% isopropanol to reduce non-specific stains, nuclei were counterstained with Mayer’s haematoxylin (Sigma). Calcium deposits were stained for with Alizarin Red S solution (2% w/v in distilled water, pH 4.2; Alfa Aesar). Sulphated proteoglycans were stained using Alcian Blue solution (1% w/v in 0.1 N HCl; Alfa Aesar). The resulting stains were observed on an Olympus CKX41 microscope using 10x and 40x objectives, images were captured with QCapture Pro software.

### Flow cytometry

Cells were harvested by trypsinisation, centrifuged at 150 x g and resuspended in phosphate buffered saline (PBS). Non-specific binding was prevented by a 10% normal goat serum (NGS, Sigma) and 0.1% sodium azide incubation on ice. Primary antibodies were added to 1x10^5^ cells per condition added for 30 minutes at room temperature. The antibodies used were against CD29 (ab52971, Abcam), CD44 (ab6124, Abcam), CD45 (ab10559, Abcam), CD73 (AD2, Miltenyi), CD90 (ab23894, Abcam), CD105 (43A4E1, Miltenyi) or relevant isotype controls. The cells were washed and incubated with the secondary antibodies (Cy5 anti-mouse or anti-rabbit, Eurogentec/Abcam) for 30 minutes at room temperature. The cells were washed, resuspended in 500 μl PBS with 3% NGS and analysed on a Beckman Coulter Cyan ADP with 635nm excitation. Fluorescence compensation and post-acquisition gating was performed using Summit V4.3.

### Propidium iodide analysis

Cells were harvested by trypsinisation, centrifuged and resuspended to 2.5x10^2^ cells/ml in 70% ethanol whilst vortexing. Cells were fixed in ethanol at 4°C for a minimum of 4 hours. The cell suspension was centrifuged and cells washed in PBS. They were then resuspended in PBS with 2.5 μg/ml RNase (Invitrogen) and 40 μg/ml propidium iodide (Invitrogen). The cells were analysed on a Beckman Coulter Cyan ADP with 635nm excitation. The percentage of cells in each cell cycle phase was analysed on Modfit LT (Verity Software House) after gating by forward and side scatter, and by pulse height and area (doublet discrimination). Statistical analysis was by one way ANOVA with Bonferroni post-hoc test separately for each phase.

### Immunocytochemistry (ICC)

Cells were cultured on glass coverslips until of a suitable density and were fixed in 4% paraformaldehyde. The cells were permeabilised in 0.3% Triton x-100 and non-specific staining was prevented by incubation in 10% goat serum in PBS. The primary antibody, p27^Kip1^ (C-19, Santa Cruz) was applied for 1 hour at room temperature, at a concentration of 1:100. The cells were washed and Cy2-conjugated anti-rabbit antibody (Jackson) was applied for 1 hour at room temperature, at a concentration of 1:200. The cells were washed and the coverslips were mounted using ProLong Gold Antifade reagent (Life Technologies) with DAPI. Slides were viewed using an Olympus BX51 upright microscope using a 40x 0.75 UPlanFLN objective and captured using a Coolsnap EZ camera (Photometrics) through MetaVue Software (Molecular Devices). Filter sets for DAPI (31000v2) and FITC (41001) were used. Images were processed and analysed using ImageJ (http://rsb.info.nih.gov/ij). To quantify nuclear stains of p27^Kip1^, 5 random fields were imaged and the colour channels were separated. A minimum total of 1000 nuclei were counted per cell line (based on DAPI staining).

### Western blotting

Protein was collected in RIPA buffer with protease inhibitors (Promega). The protein concentration was assessed using Bradford reagant (Sigma) and 20 μg of total protein was separated on a denaturing poly-acrylamide stacking gel (BioRad) by SDS electrophoresis. Protein from the gel was transferred to a methanol activated polyvinylidene fluoride (PVDF) membrane (BioRad) by electroblotting. Non-specific binding was prevented by incubation in 5% (w/v) milk powder (Sigma). The primary antibody was diluted in blocking solution for an overnight incubation at 4°C. Antibodies used were against β-actin (1:5000, Cell Signalling Technology), CyclinB1 (D-11, 1:150, Santa Cruz), CDK1 (#9116, 1:1000, Cell Signalling Technology), phospho-CDK1 (#9111, 1:1000, Cell Signalling Technology) and p27^Kip1^ (C-19, 1:200, Santa Cruz). The membrane was incubated in the HRP conjugated secondary antibody (Cell Signalling Technology) at a concentration of 1:10,000 in blocking solution for one hour at room temperature. Bound antibodies were visualised using the enhanced chemiluminescent (ECL) system (HRP Substrate, Millipore) and exposure to Biomax XAR film (Kodak). Densitometry analysis was performed using ImageJ, with normalisation to the housekeeping protein β-actin, followed by either the adult control or to standard glucose/standard insulin as appropriate. Both the p27^Kip1^ and CyclinB1 antibodies were polyclonal and showed non-specific bands as per the manufacturer’s validations, the correct bands were readily identified at the known molecular weight.

### Statistical analysis

Statistical analysis was carried out in Prism (GraphPad software) using unpaired t-tests or Mann-Whitney tests as appropriate. The results are presented as mean ± standard error of the mean, of at least 3 experiments and were considered significantly different from each other when p < 0.05.

## Results

### MSCs were derived from three CHI patients

**Fig 1:**
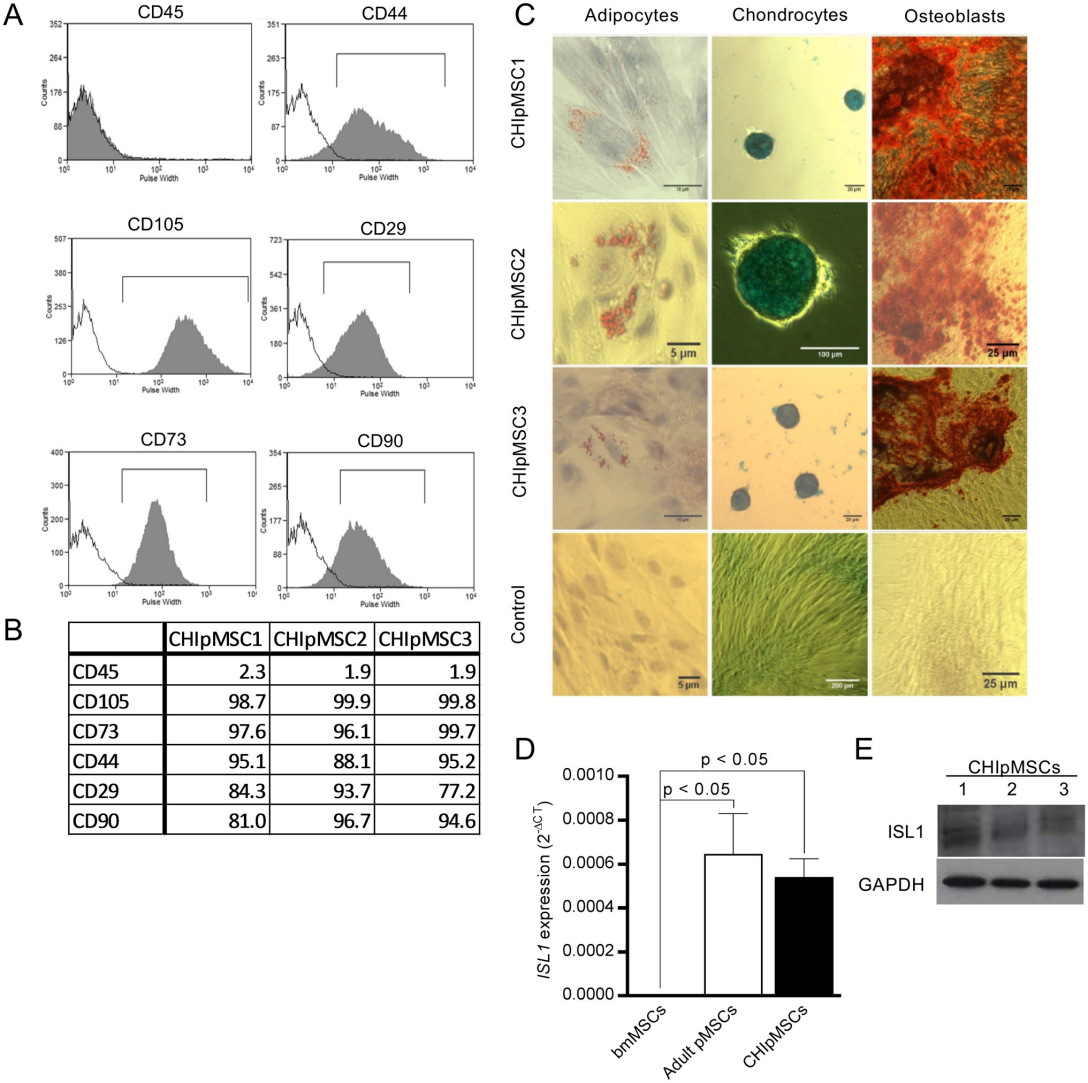
Pancreatic MSCs were derived from CHI patient tissue. **(A)** Flow cytometry showed the cells to be positive for the MSC markers CD29, CD44, CD73, CD90 and CD105 and negative for the haematopoeitic marker CD45 (shown in grey) compared to isotype controls (shown in white). Histograms show the pulse width against cell number for CHIpMSC2 and are representative of all 3 cell lines. **(B)** Percentage positive cells shown by flow cytometry for MSC markers for all CHIpMSC lines, gated as in A. **(C)** MSCs were differentiated to the three mesodermal lineages: fat, cartilage and bone, as confirmed by oil red O, alcian blue and alizarin red stains respectively, confined to the differentiated cells. **(D)** Pancreatic MSCs, both CHI and adult uniquely express the pancreatic lineage gene *ISL1*, *ISL1* could not be detected in bmMSCs by qRT-PCR. Bars represent the mean 2^-ΔCT^ with SEM from 3 unique lines for each bmMSCs, adult pMSCs and CHIpMSCs, relative to the 2 housekeeping controls. **(E)** ISL1 protein was also detected in the CHIpMSC lines by western blot.

Cell lines were derived from the excised pancreatic tissue of three patients with CHI (Table 1 for clinical details). Morphologically, the cells grown from the pancreas explants resembled MSCs and exhibited plastic adherence [21] (S1 Fig). Derived CHIpMSCs were highly positive for International Society for Cellular Therapy-defined MSC markers [21] CD29, CD44, CD73, CD90 and CD105 but only expressed very low levels of CD45, confirming an absence of contaminating blood cells from the tissue (Fig 1A and 1B).

Multipotency is an important defining characteristic of MSCs and was assessed by the ability of the MSCs to differentiate to three mesoderm derived mature cell types—adipocytes, osteoblasts and chondrocytes (Fig 1C). The differentiated cells were assessed via phenotypic assays utilising histological stains. There was widespread positive staining seen for sulfated proteoglycans in all micromass chondrogenesis cultures and for calcium in the osteogenesis cultures. Only rare positive stains were seen in the adipogenesis cultures, <1% of cells contained lipid droplets, but those which did were packed full of lipid droplets; see also [22]. No positive stains were seen in the experimental controls for calcium or lipids, slight staining was seen throughout the controls for the proteoglycan staining, but this was less intense than in the differentiated cells. The three CHI derived cell lines showed plastic adherence, MSC-specific cell surface markers and the ability to differentiate to three different cell types. Together these data confirm that the derived cells were MSCs.

In parallel, MSCs were derived from adult pancreas and characterised using similar methods (S1 Fig). These cells proliferated very slowly and stopped expanding within 20 population doublings which limited the quantity of data which could be generated.

As CHIpMSC cells are pancreatic in origin, we tested expression of a number of genes associated with pancreas development. We observed expression of *SOX9*, *ISL1*, *PAX6*, *MAFB* and *CK19* (S2 Fig), which are implicated in the development of the pancreas, but they also have other roles in different tissues. The expression of these genes was assessed in three independent bmMSC lines. All of these pancreatic development genes were expressed in the bmMSCs except for *ISL1*, which was strongly expressed in both CHI and adult control pMSCs, but was not detected in the bmMSCs, highlighting the fact that the pMSCs have a remnant feature of their tissue of origin (Fig 1D). We further confirmed expression of ISL1 protein in the CHIpMSCs. The tissue of origin was confirmed by the fact that the cell population maintained expression of mature, differentiated pancreatic cell types (*INS*, *GCG*, *SST* and *PDX1)* for the first 2–4 passages (S2C Fig), indicative of a mixed endocrine starting cell population.

### Increased Ki67 expression in CHI tissue and proliferation of CHI-derived pMSCs

It has been previously reported that the diffuse form of CHI features high rates of proliferation throughout all parts of the pancreas, including in the normally non-proliferative endocrine cells [10, 12].

**Fig 2:**
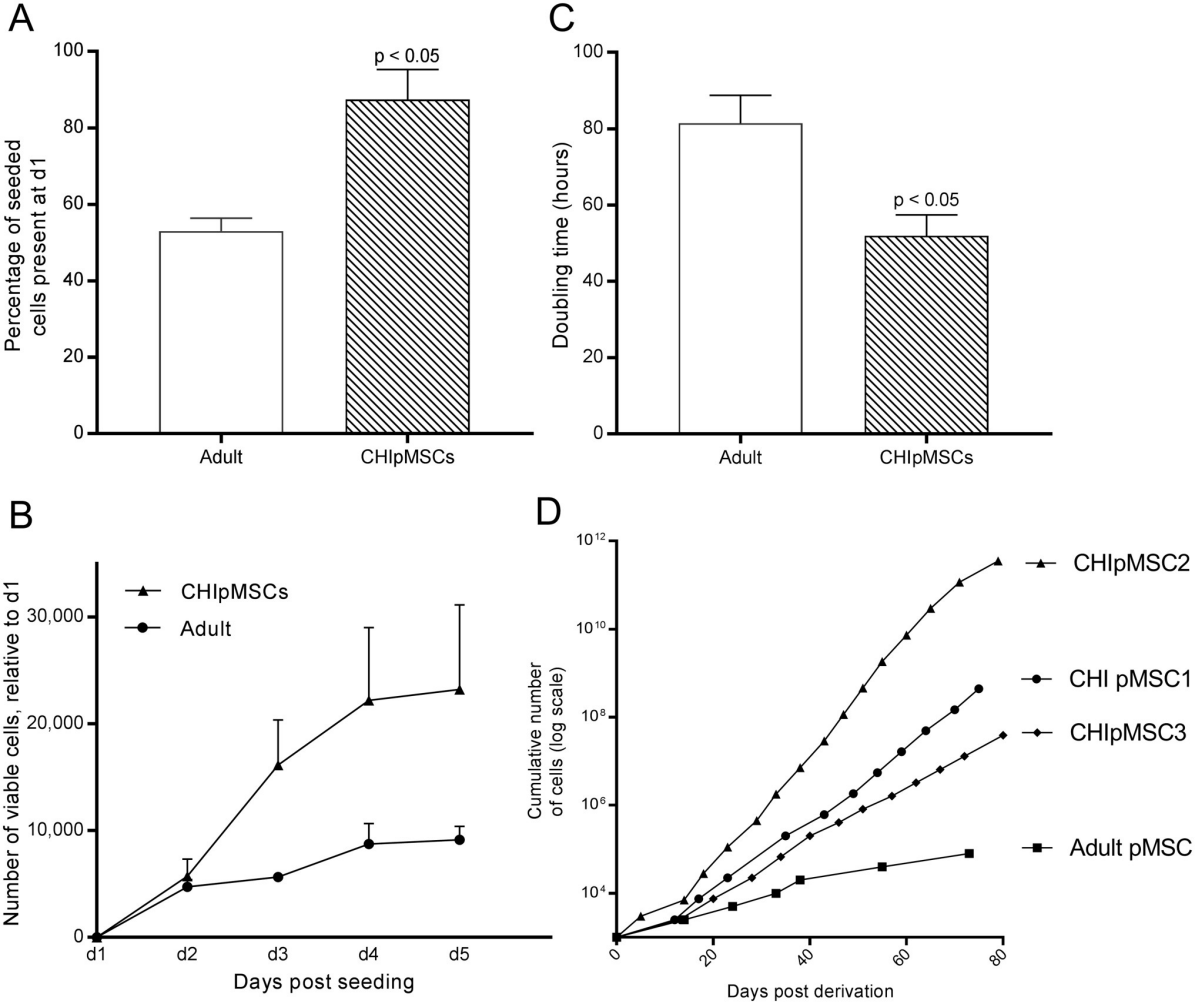
CHIpMSCs show increased proliferation *in vitro*. **(A)** An increased number of cells were present at day 1 post seeding in the CHIpMSCs compared to the adult MSCs. **(B)** Growth curves of the cultured CHIpMSCs showed increased proliferation in all three lines compared to adult control pMSCs, each point represents the mean number of viable cells at each time point, relative to the number of cells counted at day 1, with standard deviation. **(C)** The doubling time in hours of each cell line was calculated from the growth curves used for panel B and was lower in the CHIpMSCs. **(D)** The cumulative cell growth was approximated from the split ratio at each passage for the CHIpMSCs and adult MSCs, assuming an initial population of 1000 cells. Each data point represents a passage event. The last data point represents the final passage prior to cells failing to reach >60% confluency. For **(A-C)** the mean of the 3 adult MSC lines and 3 CHIpMSC lines is shown, for (**D**) one adult line is shown, growth of all 3 was similar.

Given the increased levels of Ki67 in CHI tissue, we assessed proliferation rates in the derived CHIpMSC lines via growth curves and doubling times (Fig 2). Following seeding of cells on day 0, lower numbers of cells were recovered at day 1 in adult pMSCs compared with CHIpMSC cells (Fig 2A); the differences in numbers of cells recovered at day 1 could be due to improved adherence, less cell death post passage, faster recovery or increased proliferation, or a combination of these factors. Doubling times were significantly reduced for CHIpMSCs, relative to adult pMSCs (Fig 2B and 2C). The higher rates of proliferation that we observed in all CHIpMSC lines were more striking over the long term as shown in Fig 2D. Whilst the CHIpMSC and adult pMSCs ceased to grow–failing to reach >60% confluency within 14 days post-passage—after approximately 80 days, the CHIpMSCs had undergone considerably more passages and population doublings in that time.

### Accelerated cell cycle and G_2_/M transition in CHIpMSC cells

**Fig 3:**
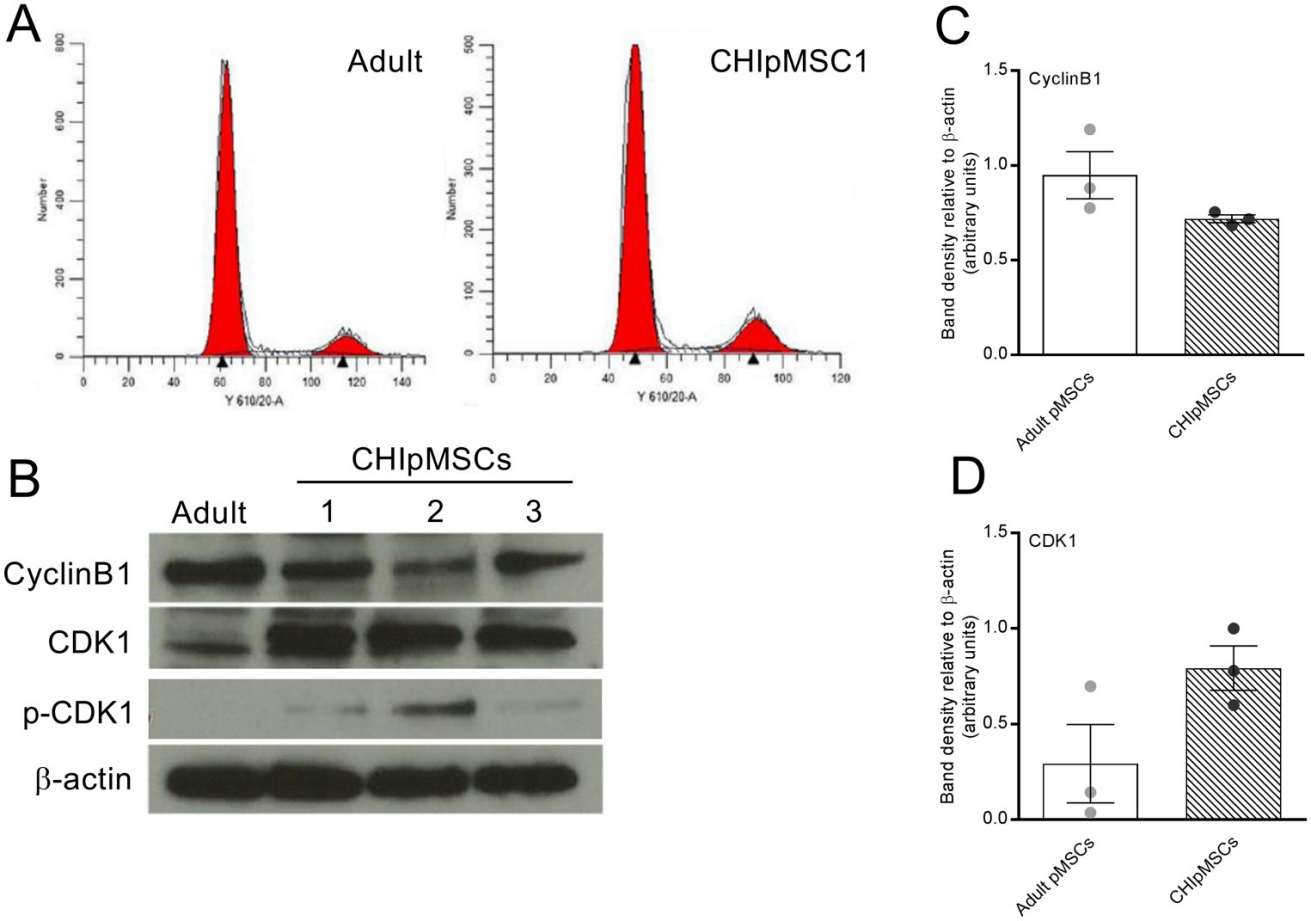
Progression through mitosis may be involved in hyperproliferation seen in CHI. **(A)** Cell cycle analysis by propidium iodide flow cytometry showed no major differences in the percentage of cells at each phase of the cell cycle between the CHIpMSCs and adult MSCs, representative plots of the flow cytometry data with quantification overlay used by Modfit LT are shown. **(B)** Western blot showed CyclinB1 to be largely stable, with its binding partner CDK1 upregulated in the three CHIpMSC lines. Phosphorylated CDK1 was detected in the CHIpMSCs but we could not accurately quantify it. **(C-D)** Densitometry analysis of western blots showed CDK1 to be upregulated **(C)** and CyclinB1 to be stable **(D)** in the CHIpMSCs compared to the adult pMSCs. The bars show the mean band density of three experiments for each of the three CHIpMSC lines compared to the mean of the three adult pMSClines relative to the loading control, with SEM, the circles represent each individual line. There was high variability in the adult pMSCs for CDK1 **(C)** and so the difference was not significant.

The differences in proliferation rates between adult-derived and CHIpMSCs could be related to either the length of the cell cycle or the proportion of cells in G_0_/G_1_. We analysed the cell cycle stages in each cell line using propidium iodide and flow cytometric analysis. Minimal differences were seen between the CHI and adult MSCs in the percentage of cells in each stage of the cell cycle, three experiments were analysed and quantified for each cell line with representative plots shown in Fig 3A. The mean percentage of cells in G_0_/G_1_ was between 78.8 and 83.8%, for S-phase was between 5.8 and 12.1% and for G_2_/M-phase was between 8.9 and 13.3% for all adult and CHIpMSCs. This suggests that high rates of proliferation are not due to a decreased percentage of cells in G_0_ but are instead due to accelerated progression of cycling cells through the cell cycle, at all stages.

To further explore regulation of cell-cycle progression, protein expression levels of the primary G_2_/M regulators cyclinB1 and CDK1 were investigated using western blot analysis (Fig 3B). CyclinB1 showed some variation but no overall changes between the CHI-derived and adult pMSCs, across three experiments (Fig 3C), however, CDK1 was more variable. Overall, the levels of total CDK1 were increased in the three CHIpMSC lines compared with 2 of the 3 adult pMSC lines, with averages of all three shown in Fig 3D. In the third experiment (i.e. with the third adult control cell line) the levels of CDK1 were similar between the adult control cells and the CHI-derived cells. All adult cell lines were derived in the same way, from cadaveric donors aged above 40, and all three experiments were carried out on adult cells between passages 4 and 10 (as with the proliferation and cell cycle experiments) and so it is unclear why this adult line was different to the other two. If this adult cell line was excluded from analysis, CDK1 was 14-fold higher in both CHIpMSC1 and CHIpMSC3 compared to the adult lines, and 24-fold higher in CHIpMSC2 compared to the adult MSCs. This correlates closely with the relative proliferation rates seen in the three cell lines—where CHIpMSC1 and CHIpMSC3 were similar and CHIpMSC2 was almost twice as fast. Further adult MSC samples would be required to confirm or refute this observation. The phosphorylated form of CDK1 was present when tested, as shown in Fig 3B, but due to the very low levels of total CDK1 present in the adult lines we could not adequately quantify if the ratio of phosphorylated to unphosphorylated CDK1 was altered.

### Cell cycle entry is controlled by p27^Kip1^ in CHI cells

**Fig 4:**
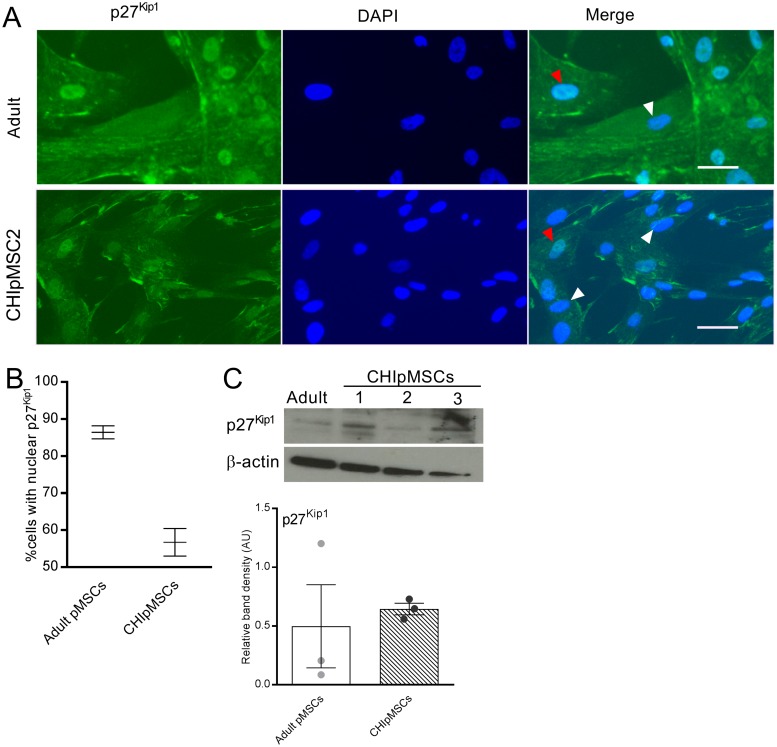
Reduced p27^Kip1^ nuclear localisation correlates with increased proliferation in CHIpMSCs. **(A)** p27^Kip1^ is localised to both the nucleus and cytoplasm in some cells, indicated by the red arrows and to the cytoplasm alone in others, indicated by the white arrows. This was the case in all cell lines studied. The scale bar represents 25 μm. **(B)** A lower percentage of CHIpMSCs had nuclear localised p27^Kip1^ than adult pMSCs. Bars show the mean with SEM, of the 3 CHIpMSC lines and the 3 lines for adult. **(C)** Total p27^Kip1^ protein was variable in the adult pMSCs but appeared to be increased.

In order to understand the mechanism by which CHIpMSCs proliferate at an increased rate, we also assessed G_1_/S cell cycle regulators which had previously been implicated in diffuse CHI [12]. CDK6, a promoter of cell cycling which shows increased nuclear localisation in CHI β-cells, showed little nuclear localisation in the CHIpMSCs (S3 Fig) suggesting it is not a key regulator in these cells and CDK4 may be fulfilling this role instead as the two show redundancy [23]. The cell cycle chaperone and/or inhibitor p27^Kip1^ was also shown to have increased nuclear localisation in CHI. In both the adult and CHIpMSCs p27^Kip1^ was split between solely cytoplasmic, and both nuclear and cytoplasmic localisation, with a consistently lower level of nuclear localisation in the CHIpMSCs (Fig 4). Conversely, the total amount of p27^Kip1^ present appeared variable, but generally increased in the CHIpMSCs–as with CDK1, but not CyclinB1, one adult donor was outlying (Fig 4C).

**Fig 5:**
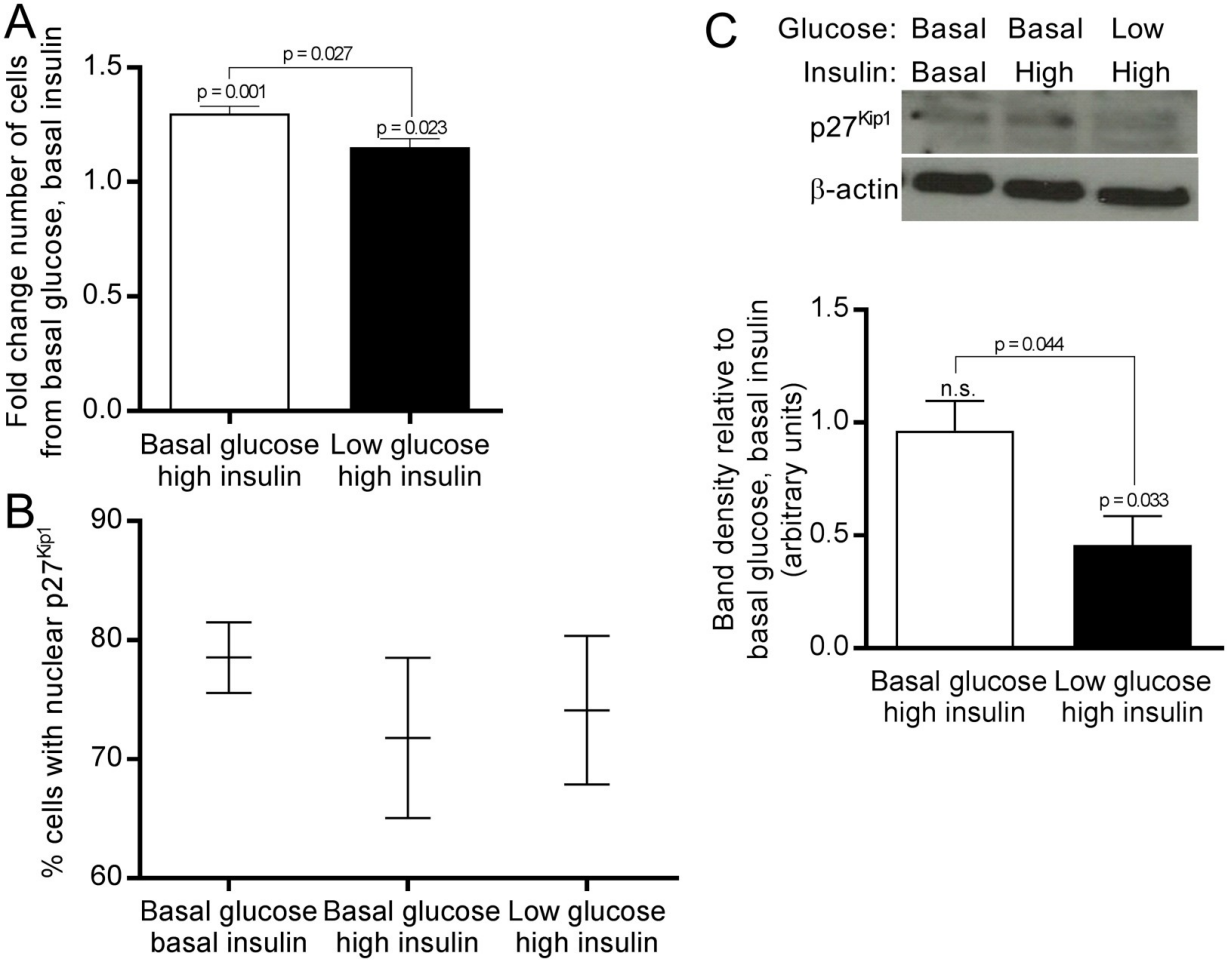
Levels and localisation of p27^Kip1^ are further influenced by high insulin and low glucose conditions seen in CHI. **(A**) Insulin induced proliferation of CHIpMSCs, even in a minimal glucose background. Bars show the mean of all three CHIpMSC lines as a fold change against the basal glucose and basal insulin condition, with standard deviation. **(B)** The percentage nuclear localisation of p27^Kip1^ was variable across the three CHIpMSC lines, but showed a trend of nuclear localisation which was the inverse of the number of cells. **(C)** Western blot showed total levels of p27^Kip1^ were not affected by increased insulin but were lowered by low glucose, confirmed by densitometry analysis, bars represent the mean from each of the three CHIpMSC lines with standard deviation.

To investigate why nuclear p27^Kip1^ was increased in diffuse CHI tissue, but decreased in the CHIpMSC cells we manipulated the glucose and insulin concentrations in cell culture media to simulate *in vivo* CHI conditions. As expected we found insulin to have a mitogenic effect on the CHI cell lines. When glucose levels were lowered, insulin was still able to drive higher rates of proliferation although not as strongly as with physiological levels of glucose, shown in Fig 5A. This suggests that the increased insulin levels in the CHI pancreas cause increased proliferation despite lowered glucose levels. We found that p27^Kip1^ was affected by altering both the glucose and insulin levels. Looking at the distribution patterns of p27^Kip1^ in these conditions, there was a trend of decreased nuclear p27^Kip1^ when insulin levels were increased, but a slightly higher percentage of nuclei stained in the lower glucose condition than standard glucose. This trend which is shown in Fig 5B, is present in each line and is the inverse of the cell numbers. Total levels of p27^Kip1^ were unchanged by increasing the insulin levels, so this was not the method by which the increased proliferation was driven. Further, there was a dramatic decrease in the total levels of p27^Kip1^ in response to decreasing the glucose levels in all three cell lines.

Together these data suggest that increasing insulin causes p27^Kip1^ to be sequestered in the cytoplasm thereby allowing cell cycle progression, either by translocation from the nucleus to the cytoplasm or inhibition of shuttling to the nucleus. The hypoglycaemia caused by hyperinsulinaemia in the patients likely causes a decrease in the total amount of p27^Kip1^ protein in the CHI pancreas, which in turn affects the dynamic regulation of protein location.

## Discussion

In this study we report the reproducible derivation of cell lines from the pancreatic tissue of three patients with CHI, designated CHIpMSC1-3. Each patient had a different form of the disease which had no impact on the ability to derive a cell line. The cell lines were identified as MSCs by plastic adherence, multipotency and expression of cell surface markers [21]. CHIpMSC cells expressed a number of genes associated with pancreatic development, all of which were also expressed in bmMSCs except for *ISL1*. Some of these markers were similar to those reported before in pMSCs [24], although another study showed pMSCs to be *ISL1* negative [25]. However this suggests that CHIpMSCs maintain some features of their pancreatic origins following de-differentiation in culture [22, 26, 27].

CHI is a disease which features increased rates of proliferation, along with increased nuclear localisation of two G_1_/S-phase regulators [12]. The precise mechanism of this increased proliferation has not yet been elucidated, but may be related to the increased levels of insulin acting as a mitogen. In line with the increased proliferation seen in the pancreas of CHI patients, we observed increased proliferation rates in CHIpMSCs when compared to adult control pMSCs under identical culture conditions demonstrating underlying differences in the cells outside of the immediate mitogenic effect of insulin. Cell type differences in MSCs have been previously seen, both in proliferation rate and longevity in culture supporting our observation that the phenotype is maintained *in vitro* [20]. Differences in proliferation rates could be partially due to the age of the patients—unfixed neonatal control tissue was not available. However, the rates of proliferation between different CHIpMSC lines was not correlated to the individual donor patients’ ages with the CHIpMSC2 line showing the fastest proliferation despite the patient being over 1 year older than the other two. Increased proliferation may be due to increased entry to the cell cycle. Cell cycle analysis did not show conclusive evidence of this, but in contrast we saw decreased nuclear expression of the G_1_/S regulator p27^Kip1^ in the CHI cells compared to the adult cells.

Previously *in vivo* it was shown that there is increased p27^Kip1^ nuclear localisation in CHI cells. It was suggested that this may contribute to proliferation through its function as a chaperone for CDKs [28]. However, Ki67 co-localised with CDK6 but not p27^Kip1^ in the tissue; co-localisation with Ki67 and CDK6 would be expected if p27^Kip1^ were assembling the G_1_/S promoting molecules. By stimulating the cells with insulin, as occurs in CHI, we aimed to recapitulate the *in vivo* conditions, but in fact saw further decreases in nuclear p27^Kip1^ after four days, reflecting the increased proliferation. When lowering glucose levels to further simulate the CHI condition, there was a significant decrease in total p27^Kip1^ which began to reverse the alterations to its localisation. This mirrors the findings of Zhang et al. [29], who found that increasing glucose concentration caused an increase in total p27^Kip1^, although this decreased proliferation, pointing to a role for glucose in regulation of the total levels of protein which in turn affects localisation and shuttling. With an acute stimulus *in vitro* after culture in healthy glucose/insulin levels we can see a mild effect on p27^Kip1^ levels and location, with the chronically abnormal levels in the patients this is inevitably more striking. The combined interaction of both glucose and insulin on p27^Kip1^ regulation and dynamic shuttling, and the mechanisms thereof are unknown but may help us to understand the proliferation seen in CHI.

G_2_/M regulators have not been studied within the CHI pancreas previously. The differences seen in the rate of the cell cycle could also be accounted for by increased progression through M-phase so we investigated the primary promoters of entry to M-phase—CDK1 and CyclinB1 which act together as a complex. CDK1 is expressed stably throughout the cell cycle, whilst cyclinB1 is degraded for M-phase exit [30], but the majority of regulation is post-translational—by sub-cellular localisation and phosphorylation of CDK1. CyclinB1 was expressed at a consistent level across all the CHI and adult cell lines, but its partner, CDK1 was expressed at a much higher level in the CHIpMSCs compared to two of three adult lines. Going forward, it would also be beneficial to assess CDK1 in post-operative CHI tissue in line with previous studies to confirm whether CDK1 is involved with hyperproliferation in CHI.

Chronic exposure to high insulin and low glucose prior to the cell lines being derived into baseline conditions has caused the cells to maintain a hyperproliferative profile, despite removing the stimuli. This observation points to potential genetic or epigenetic alterations, indeed high glucose levels have recently been shown to alter the methylome of human β-cells [31]. CDK1 is also known to be associated with DNA damage [32], with double stranded DNA breaks previously observed in CHI triggering the p53 pathway [33]. As such the increased proliferation we continue to see is perhaps due to continued deregulation of cell cycle proteins due to a background of DNA damage which has implications for the health of CHI patients who do not undergo surgery.

In conclusion we have developed a pancreatic specific MSC model from patients with CHI which demonstrates increased rates of proliferation, similar to that observed in patients. Increased proliferation seems to be due in part to an increased progression of cells through the cell cycle with a decrease in nuclear p27^Kip1^ and an increase in total CDK1. The cells respond dynamically to glucose and insulin as expected, with proliferation changes similar to β-cells *in vivo* [34, 35]. Both glucose and insulin affected p27^Kip1^ but in different ways, the interplay between these two stimuli and the resulting effects on p27^Kip1^ may help to explain some aspects of the pathology of CHI. We therefore propose CHIpMSCs as a novel *in vitro* model which can be manipulated to understand the increased proliferation seen in CHI.

## Supporting information

S1 Fig Adult and CHIpMSCs were phenotypically confirmed as MSCs. **(A)** Adult pMSCs showed plastic adherence and a normal morphology for MSCs, the scale bar represents 200 μm. **(B)** A subset of cell surface markers were assessed by flow cytometry, adult pMSCs were overall positive for CD44, CD73 and CD90, and negative for CD45, compared to isotype controls, similar to the CHIpMSCs. **(C)** CHIpMSCs showed mesenchymal morphology and plastic adherence.
(TIF)

S2 Fig Gene expression analysis showed a pancreatic signature in CHIpMSCs. **(A)** Expression of the pancreatic development associated genes *SOX9*, *ISL1*, *PAX6*, *MAFB* and *CK19* was observed in all three CHIpMSC lines by RT-PCR throughout their span in culture, shown here at passage 9. **(B)** Expression of all of the genes shown in panel A was also observed in three independent bmMSC lines suggesting that in this context the markers are of MSCs not pancreas development, except for *ISL1* which was not detected by RT-PCR in the bmMSCs. **(C)** Expression of the pancreatic islet genes *PDX1*, *INS*, *GCG* and *SST* was detected by RT-PCR in the early cultured cells, shown here at passage 2, highlighting their pancreatic origin, but expression of these genes was subsequently lost.
(TIF)

S3 Fig CDK6 staining was dispersed throughout the nucleus and cytoplasm. Immunostaining for CDK6 did not show a clear difference between cytoplasmic and nuclear localisation, shown here in CHIpMSC3, a similar staining pattern was seen for all CHIpMSCs and adult pMSCs.
(TIF)
