# Correction: Increased proliferation and altered cell cycle regulation in pancreatic stem cells derived from patients with congenital hyperinsulinism

**DOI:** 10.1371/journal.pone.0223999

**Published:** 2019-10-10

**Authors:** Sophie G. Kellaway, Karolina Mosinska, Zainaba Mohamed, Alexander Ryan, Stephen Richardson, Melanie Newbould, Indraneel Banerjee, Mark J. Dunne, Karen E. Cosgrove

The NC3Rs grant number is missing from the Funding statement. The correct Funding Statement is as follows: This work was funded by NC3Rs (https://www.nc3rs.org.uk/, SGK awarded to MJD, KC, NC/K500306/1), and Diabetes UK (https://www.diabetes.org.uk/, KM awarded to MJD, KC). AR was funded by Heptares/Innovate UK (https://soseiheptares.com/ awarded to MJD). KEC was funded by a Research Councils UK Academic Fellowship (https://www.ukri.org/). The funders had no role in study design, data collection and analysis, decision to publish, or preparation of the manuscript.
