# Supplementary figures and images for: Increased proliferation and altered cell cycle regulation in pancreatic stem cells derived from patients with congenital hyperinsulinism
Source: PLoS One. 2019 Sep 16;14(9):e0222350. doi: 10.1371/journal.pone.0222350 (PMC6746350; doi:10.1371/journal.pone.0222350)

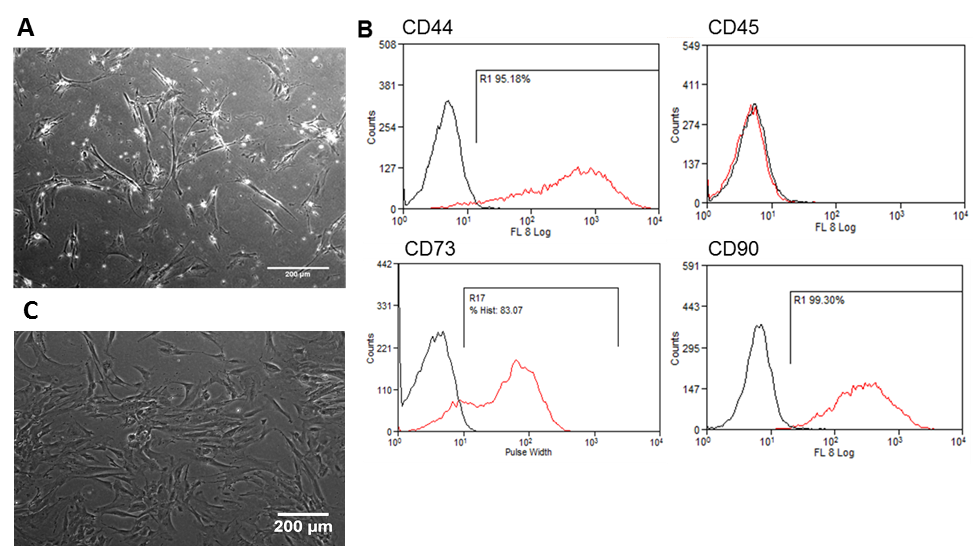

Supplement: S1 Fig — (A) Adult pMSCs showed plastic adherence and a normal morphology for MSCs, the scale bar represents 200 μm. (B) A subset of cell surface markers were assessed by flow cytometry, adult pMSCs were overall positive for CD44, CD73 and CD90, and negative for CD45, compared to isotype controls, similar to the CHIpMSCs. (C) CHIpMSCs showed mesenchymal morphology and plastic adherence. (TIF) [file pone.0222350.s001.tif]

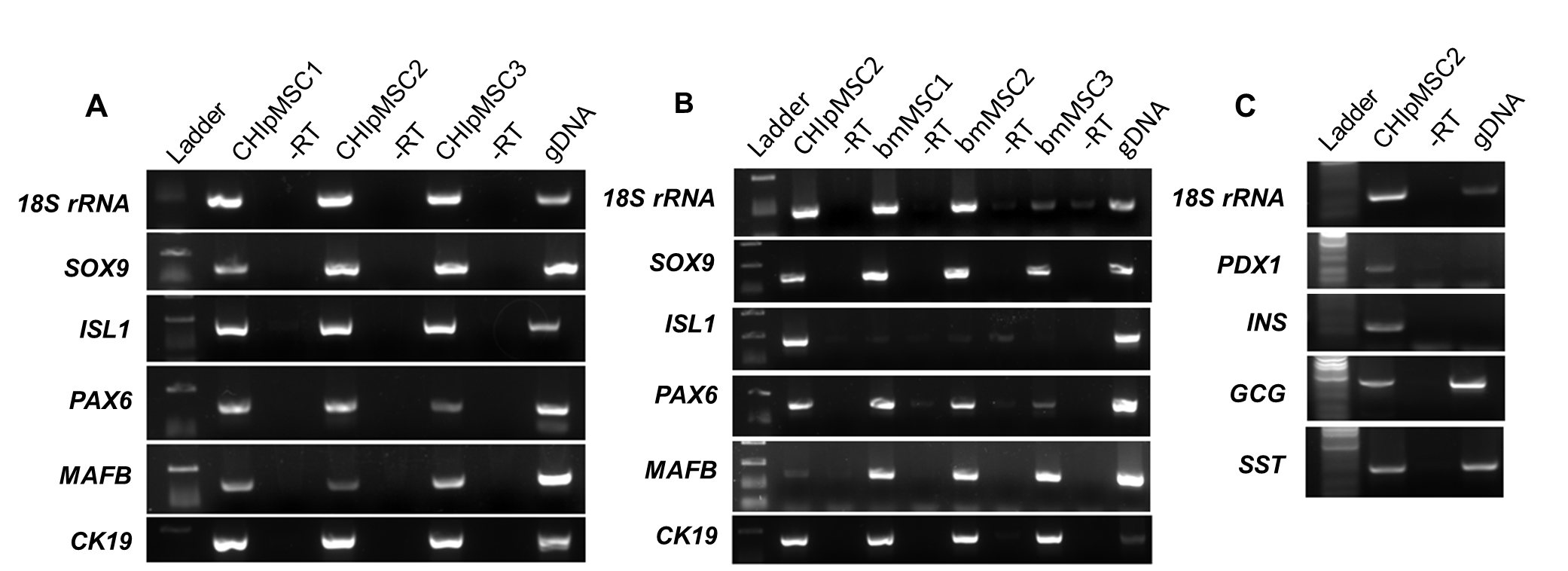

Supplement: S2 Fig — (A) Expression of the pancreatic development associated genes SOX9, ISL1, PAX6, MAFB and CK19 was observed in all three CHIpMSC lines by RT-PCR throughout their span in culture, shown here at passage 9. (B) Expression of all of the genes shown in panel A was also observed in three independent bmMSC lines suggesting that in this context the markers are of MSCs not pancreas development, except for ISL1 which was not detected by RT-PCR in the bmMSCs. (C) Expression of the pancreatic islet genes PDX1, INS, GCG and SST was detected by RT-PCR in the early cultured cells, shown here at passage 2, highlighting their pancreatic origin, but expression of these genes was subsequently lost. (TIF) [file pone.0222350.s002.tif]

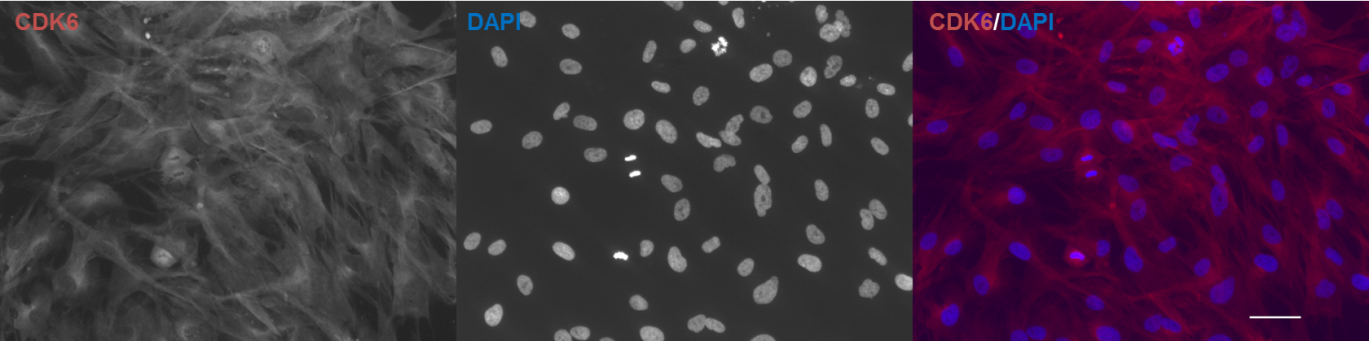

Supplement: S3 Fig — Immunostaining for CDK6 did not show a clear difference between cytoplasmic and nuclear localisation, shown here in CHIpMSC3, a similar staining pattern was seen for all CHIpMSCs and adult pMSCs. (TIF) [file pone.0222350.s003.tif]
